# Editorial: The role of emotional dysregulation in addiction

**DOI:** 10.3389/fpsyg.2023.1253541

**Published:** 2023-07-28

**Authors:** Paul J. Meyer, Gabriel Segal

**Affiliations:** ^1^Department of Psychology, University at Buffalo, Buffalo, NY, United States; ^2^King's College London, London, United Kingdom

**Keywords:** substance use disorder, addiction, emotional dysregulation, philosophy, social media, circadian rhythms, social stress, alcoholism

## Introduction

Drugs, including alcohol, stimulants, opiates, and cannabis, have the ability to influence emotions by either alleviating negative emotions or intensifying positive ones. For instance, individuals may turn to alcohol as a means of relaxation during stressful periods or to enhance their enjoyment of positive experiences. Moreover, stress is widely recognized as a significant factor in vulnerability to relapse. Addiction therapists often educate their clients about the use of drugs to alter emotions, and it can be argued that various therapeutic approaches, such as abstinence programs like the 12-step model, assist individuals in recovery by helping them effectively manage their emotions and cope with stress. Furthermore, research suggests that young individuals at a higher risk of addiction exhibit hypersensitivity in brain systems that strongly respond to emotionally significant stimuli, such as stress and the subjective effects of drugs. These same systems also respond physiologically to drugs.

The objective of this Research Topic was to investigate the involvement of emotional dysregulation and stress in the development of addiction and relapse. One hypothesis proposes that heightened sensitivity to emotions makes drugs appealing to young individuals, as they seek to intensify positive feelings and alleviate negative ones. Over time, repeated drug use leads to an exaggerated response from the body toward drugs and associated cues, ultimately resulting in addiction. However, the evidence supporting this hypothesis is inconclusive, with mixed findings from research studies.

## The contributions

Although the four articles that contributed to this Research Topic differed in their specific research focus, subject populations, and contexts, they shared the overarching theme of investigating emotional dysregulation and addiction, providing valuable insights into understanding the factors and consequences associated with addictive behaviors.

In his paper “*Addiction and autonomy: Why emotional dysregulation in addiction impairs autonomy and why it matters*” Henden directly tackles the role of emotional dysregulation and argues that it is a factor contributing to the loss of control and impaired autonomy experienced by individuals with substance use disorder. He distinguishes between reflective and behavioral self-control, with the latter involving adaptive behavioral regulation in the face of emotional challenges. However, when emotional regulation becomes maladaptive, it leads to emotional dysregulation. In such cases, drug use can serve as a means of emotion regulation, and when it becomes more frequent, it can lead to a “crowding out” effect where drug-related emotions dominate the individual's practical perspective. This can result in a loss of interest in other activities and a decrease in the emotional salience of non-drug-related experiences. Emotional dysregulation can contribute to a downward spiral of compulsive drug use and the inability to control drug intake, thus narrowing the focus on drug-oriented goals and values. He argues that this applies even in the case of voluntary drug use, i.e., in cases where drugs are used in an apparently willing and controlled fashion.

However, as described by Henden, views of decision-making that incorporate emotions and stress favor a case-based approach due to the multiple factors influencing the transition from adaptive emotional regulation to emotional dysregulation. In their paper “*The effects of circadian desynchronization on alcohol consumption and affective behavior during alcohol abstinence in female rats*,” Meyer et al. use a rodent model to investigate one such factor: whether a stressor such as circadian rhythm disruption might interact with drug use to induce emotional dysregulation. The study employs reductionist models of anxiety, such as the elevated plus-maze and marble burying tests, to examine changes in the emotional state of female rats during alcohol abstinence under circadian disruption. Overall, the study demonstrates that internal circadian desynchronization strongly affects female physiology but has only minor effects on mood-related behavior and alcohol consumption when considered separately. However, when the effects of alcohol consumption and circadian disruption are combined, changes in mood-related behaviors become more apparent. Thus, commonly encountered emotional challenges like circadian dysfunction can significantly contribute to the etiology of mood-related behavior during alcohol dependence.

In the paper “*Measuring attentional bias in smokers during and after psychosocial stress induction with a Trier Social Stress Test in virtual reality via eye tracking*” by Schröder and Mühlberger, emotional regulation is challenged in an experiment using a virtual adaptation of the Trier social stress test, where subjects are asked to perform a public speaking task or a mock job interview. Smokers and control subjects are then allowed to view scenes containing smoking-related stimuli. The findings indicate that smokers show a specific attentional bias characterized by earlier and longer fixations on smoking-related stimuli. These findings suggest that attentional bias is not a persistent trait in smokers but rather depends on the context. It is proposed that emotional learning processes, such as the association between smoking and stress relief, may contribute to changes in attentional bias, including increased initial attention and deeper processing of smoking-related stimuli.

Finally, the essential role of emotional dysregulation in addiction-related behaviors is evidenced by its role in non-drug-related behaviors as well. In their paper “*Risk Factors Associated with Social Media Addiction: An Exploratory Study*,” Zhao et al. aim to identify potential risk factors associated with social media addiction. Specifically, they investigate the influence of age, gender, impulsivity, self-esteem, emotions (anxiety, depression, social anxiety), and attentional bias using a survey of 520 college students. The findings indicate that being female, having impulsive tendencies, low self-esteem, experiencing anxiety and social anxiety, and displaying negative attentional biases increase the risk of developing social media addiction. These factors collectively account for 38% accuracy in predicting social media addiction. Consistent with Henden's perspective and the findings of Meyer et al., these results suggest that interventions targeting anxiety reduction and promoting alternative reinforcing activities could help mitigate this form of addiction.

## Conclusions, and a limitation

The contributions to this Research Topic highlight emotional dysregulation as a key feature of substance use disorder and non-drug addictions. These papers demonstrate how this dysregulation can lead to, and be facilitated by, the loss of control associated with drug use or even social media use. Thus, the manner in which this can lead to a downward spiral toward compulsive usage patterns is evident, as described in papers by Koob and Le Moal (2001). However, one principle not directly addressed by this set of articles is the role of positive reinforcement processes. For example, Robinson and Berridge (1993) argue that the incentive properties of the drug, i.e., the desire or “wanting” to take the drug, can be amplified independently from the negative reinforcement mediated by emotional dysregulation. In many cases, however, there may be a complex interaction between emotional regulation and positive reinforcement, such that reinforcing behaviors such as drug use distract from aversive emotional states (Weiss et al., 2015). It is also likely that these processes occur in tandem and to varying degrees throughout the progression of substance use disorder (Piazza and Deroche-Gamonet, 2013). However, this set of articles does not address the relationship between emotional dysregulation and positive reinforcement, which is a topic deserving further study.

## Author contributions

All authors listed have made a substantial, direct, and intellectual contribution to the work and approved it for publication.

## References

[B1] Koob G. F. Le Moal M. (2001). Drug addiction, dysregulation of reward, and allostasis. Neuropsychopharmacol. Off. Pub. Am. Coll. 24, 97–129. 10.1016/S0893-133X(00)00195-0 11120394

[B2] Piazza P. V. Deroche-Gamonet V. (2013). A multistep general theory of transition to addiction. Psychopharmacology 229, 387–413. 10.1007/s00213-013-3224-4 23963530 PMC3767888

[B3] Robinson T. E. Berridge K. C. (1993). The neural basis of drug craving: an incentive-sensitization theory of addiction. Brain Res. Rev. 18, 247–291. 10.1016/0165-0173(93)90013-P 8401595

[B4] Weiss N. H. Sullivan T. P. Tull M. T. (2015). Explicating the role of emotion dysregulation in risky behaviors: A review and synthesis of the literature with directions for future research and clinical practice. Curr. Opin. Psychol. 3, 22–29. 10.1016/j.copsyc.2015.01.013 25705711 PMC4332392

